# Prebiotics and Probiotics for Gastrointestinal Disorders

**DOI:** 10.3390/nu16060778

**Published:** 2024-03-09

**Authors:** Sameeha Rau, Andrew Gregg, Shelby Yaceczko, Berkeley Limketkai

**Affiliations:** Vatche & Tamar Manoukian Division of Digestive Diseases, David Geffen School of Medicine at University of California Los Angeles, Los Angeles, CA 90095, USA; rau.sameeha@gmail.com (S.R.); agregg@mednet.ucla.edu (A.G.); shelby.yaceczko@gmail.com (S.Y.)

**Keywords:** prebiotics, probiotics, gut microbiome, dietary supplements, short-chain fatty acids, constipation, diarrhea, irritable bowel syndrome, inflammatory bowel disease

## Abstract

The complex role of the gut microbiome in the pathogenesis of gastrointestinal (GI) disorders is an emerging area of research, and there is considerable interest in understanding how diet can alter the composition and function of the microbiome. Prebiotics and probiotics have been shown to beneficially modulate the gut microbiome, which underlies their potential for benefit in GI conditions. Formulating specific recommendations for the public regarding these dietary supplements has been difficult due to the significant heterogeneity between strains, doses, and duration of treatment investigated across studies, as well as safety concerns with administering live organisms. This review aims to summarize the existing evidence for the use of prebiotics and probiotics in various GI disorders, paying special attention to strain-specific effects that emerged and any adverse effects noted.

## 1. Introduction

The human gastrointestinal (GI) tract can crudely be defined as the hollow viscus that extends from the mouth to the anus which is responsible for the digestion and absorption of nutrients and excretion of waste products. When examined more closely, the GI tract is a highly complex, specialized, and elegant machine that is essential for survival and well-being. While the host physiology of the GI tract and its respective organs has been well studied and established, the role of the trillions of microbes, including bacteria, viruses, fungi, and protozoa, remains an intense area of investigation. These microorganisms, defined as the human microbiome, have co-evolved with humans to form diverse communities within the GI tract that are intimately involved with numerous aspects of metabolism, development of our immune system, and even regulation of our behavior [1,2]. While the human genome consists of ~23,000 genes, the gut microbiome provides an additional ~3 million or more “exogenous” genes, contributing a remarkable diversity of metabolites to enhance host function and health [3]. These metabolites produced by the microbiome, including metabolites produced by the host GI tract cells and tissues, are collectively known as the GI metabolome. The metabolome, which includes small molecules, amino acids, lipids, carbohydrates, and hormones, is highly dynamic and is influenced by diet, environmental exposures, genetics, stress, and microbial diversity. It therefore stands to reason that alterations in the microbiome and metabolome can have significant implications for human health and disease.

Alteration of the human microbiome and metabolome can be achieved through dietary changes, medications such as antibiotics, or ingestion of microbes themselves. In fact, the first suggestion of microbial ingestion as a therapeutic intervention came in 1907 when Nobel laureate Elie Metchnikoff reported the linkage between the ingestion of fermented milk with high levels of viable *Lactobacilli* and the longevity of Bulgarians [4]. Since then, the notion of possible therapeutic modulation of the microbiome and metabolome has intrigued researchers, and since the advent of DNA and RNA sequencing in the 1990s, the field has exploded.

Prebiotics and probiotics, defined in more detail below, are agents that, when ingested in adequate amounts, can influence the composition of the microbiome and metabolome, with important implications for the maintenance of healthy states, as well as the treatment of disease. This narrative review aims to (1) define prebiotics and probiotics as well as their proposed mechanism of action, (2) describe their role in inflammation and the gut metabolome, and (3) summarize the existing evidence for the use of these supplements in GI disorders.

## 2. Definitions and Mechanism of Action

### 2.1. Prebiotics

Prebiotics are non-digestible, fermentable food ingredients that alter the composition and/or activity of gastrointestinal bacteria that confer benefit to the host [5]. Most prebiotics are dietary fibers; however, not all dietary fibers have prebiotic properties [6]. The main groups of prebiotics are fructans, which include inulin and fructo-oligosaccharides (FOSs), galacto-oligosaccharides (GOSs), lactulose, resistant starch, glucose-derived oligosaccharides such as polydextrose, and pectin oligosaccharides (POSs). These compounds naturally exist in many food products, such as garlic, onion, chicory, asparagus, Jerusalem artichoke, tomatoes, wheat, barley, and rye [7]. However, given their low concentration in food products, some prebiotics are also synthetically produced on a large scale and can be added to food products [8].

### 2.2. Probiotics

Probiotics are live, non-pathogenic microorganisms that can also alter the gut microbiome, conferring host benefit [9]. They can be found in a variety of fermentable foods or purchased in the form of pills, powders, and liquid drops, and are often enteric-coated or microencapsulated to prevent destruction by gastric acid and intestinal bile salts [10,11]. Probiotic products primarily contain one or more microbial strains, typically belonging to the following genera: *Lactobacillus*, *Bifidobacterium*, *Lactococcus*, *Streptococcus*, *Enterococcus*, or *Bacillus*. Strains of yeast belonging to the genus *Saccharomyces* are also commonly used [12]. Current evidence suggests that most probiotic supplements do not colonize the host long-term (>6 months), most likely due to competition with existing host microbiota. This necessitates continued supplementation for long-term benefits but also averts the potential risk of the probiotic disrupting the surrounding microbiota or entering systemic circulation [13].

### 2.3. Synbiotics

Synbiotic products are a combination of prebiotics and probiotics that may exert a synergistic effect. The prebiotic component is thought to improve the viability of the probiotic component, as a key property of prebiotics is resistance to acids, proteases, and bile salts in the upper GI tract [12]. The combination of *Bifidobacterium* or *Lactobacillus* with FOSs is commonly used in synbiotic formulations [12].

### 2.4. Mechanism of Action

The primary therapeutic advantages of prebiotics and probiotics include (1) modulation of the host immune system and nervous system, (2) improved intestinal barrier function and nutritional absorption, (3) competition with pathogens for nutrients and adhesion to the gut mucosa, and (4) production of antimicrobial substances [12]. While the exact mechanism by which they exert these effects remains poorly understood, current evidence suggests that the production of immunomodulatory metabolites plays a significant role.

Probiotic bacteria ferment prebiotics and other dietary components to produce multiple metabolites that can alter the gut microbiome and enter systemic circulation, thus affecting other organ systems [5,8]. Among the most studied metabolites are short-chain fatty acids (SCFAs), such as acetate, propionate, and butyrate. SCFAs modulate gene transcription by inhibiting histone deacetylase activity and activating G-protein-coupled receptors (GPCRs). Through these mechanisms, SCFAs can alter colonic motility and blood flow and reduce gastrointestinal pH, which can influence nutrient absorption. Activation of specific GPCRs expressed on enteroendocrine L-cells by SCFAs can also trigger the release of gut peptides (such as GLP-1), which are involved in gut barrier function and energy metabolism [14]. Furthermore, SCFAs exert anti-inflammatory functions by modulating immune cell chemotaxis, inhibiting the release of pro-inflammatory cytokines, and stimulating the release of IgA and IL-6. Immunoregulatory probiotics can induce the release of IL-10 and regulatory T-cells, rendering them useful in autoimmune disease, allergy, IBD, and inflammation. In contrast, immunostimulatory probiotics stimulate IL-12 production, which activates helper T-cells and natural killer (NK) cells, thus boosting the response against infections or cancer cells [15,16,17]. Additionally, SCFAs promote accelerated pathogen clearance by increasing the production of reactive oxygen species. There is also evidence suggesting that butyrate may exhibit an anti-cancer effect through the induction of apoptosis and/or upregulation of a butyrate transporter [18].

Several other metabolites have also demonstrated an immunomodulatory effect on the host. For example, probiotic bacteria such as *L. reuteri* and *B. infantis* produce indole derivatives from dietary tryptophan, which can promote ILC3 cells and IL-22 production and strengthen the integrity of the intestinal mucosa via activation of the aryl hydrocarbon receptor [19,20]. Another group of metabolites, polyamines, are derived from arginine and have been shown to enhance intestinal mucosa and inhibit the expression of pro-inflammatory cytokines by lipopolysaccharide (LPS)-stimulated immune cells [19]. Some strains of *L. reuteri* have additionally been identified to produce antimicrobial compounds such as reuterin and polyketides [21]. Finally, probiotic bacteria can modify bile acids produced by the host to produce secondary bile acids, such as ursodeoxycholic acid (UCDA). A recent study demonstrated that supplementation of *L. acidophilus* with subsequent production of UCDA reduced inflammation in mice with ulcerative colitis via the activation of multiple signaling pathways and modulation of Treg cells and M1 macrophages [22].

In addition to the mechanisms described above, probiotics are thought to directly compete with pathogens for adhesion to the gut mucosa and enhance intestinal barrier function by promoting mucin production, as well as by upregulating tight junction protein expression [8,10,23,24,25,26,27]. Lastly, probiotics and prebiotics can produce neurotransmitters, which not only act locally in the enteric nervous system but also centrally [2,23,28,29,30]. These neurotransmitters include alterations in dopamine, serotonin, and gamma-aminobutyric acid (GABA).

## 3. Impact of Prebiotics and Probiotics on Intestinal Permeability, Inflammation, and Nutrient Absorption

The intestinal mucosa is a highly regulated and specialized epithelium designed to not only absorb critical nutrients and excrete toxins but also provide a physical and immunologic barrier between the microbial- and pathogen-rich lumen and the submucosal tissue and circulatory system. In healthy individuals, it is presumed that this intestinal barrier is intact, allowing for the flow of water, small molecules, and other nutrients between the intestinal lumen and systemic circulation, maintaining a homeostatic state and tempering inflammation. In diseased states, this barrier can be impaired, resulting in increased intestinal permeability, allowing pathogenic microbial components such as LPS or even entire microbes into the systemic circulation, and causing inflammatory states and malabsorption. Increased intestinal permeability has been implicated in numerous disease states including autoimmune conditions such as systemic lupus erythematosus, cardiovascular disease, obesity, fatty liver disease, and inflammatory bowel disease, to name a few [31,32,33,34,35,36,37,38,39,40]. While there are several mechanisms by which the intestinal barrier is regulated, the host microbiome and metabolome appear to be critically involved.

Prebiotics and probiotics therefore present an exciting and promising therapeutic approach to mitigate the effects of increased intestinal permeability, inflammation, and nutrient malabsorption. For instance, one study utilized high-dose aspirin to promote increased intestinal inflammation and permeability in human subjects [34]. When patients were treated with aspirin and a probiotic *Bifidobacterium* strain or prebiotic GOS, intestinal permeability was reduced, suggesting improved barrier function [34]. Another study utilizing non-steroidal anti-inflammatory drug (NSAID)-mediated inflammation in mice demonstrated increased intestinal secretion of tumor necrosis factor-alpha (TNF-α), lactate dehydrogenase (LDH), and C-reactive protein (CRP) in response to indomethacin administration [41]. Interestingly, this heightened inflammatory response was mitigated by the co-administration of two separate probiotic *Lactobacillus* strains, suggesting an anti-inflammatory effect of these probiotic strains [41]. Lastly, several studies have demonstrated improved micronutrient absorption with prebiotic and probiotic use. Specifically, in children, post-menopausal women, and geriatric patients, probiotic supplementation improved serum calcium concentration when compared to placebo controls, suggesting improved absorption within the intestinal lumen, possibly due to SCFAs affecting colonic pH and enhancing calcium solubility [42,43,44,45]. Supplementation of prebiotic GOSs was found to increase calcium absorption in postmenopausal women and adolescent girls, thought to be mediated by increased *Bifidobacteria* levels [46,47]. Additionally, some probiotics are natural producers of B vitamins [10,12].

## 4. Methods

A search query was designed to capture articles pertaining to the use of pre- or probiotics in the context of gastrointestinal disorders (Supplementary Material 1). A list of 8506 potentially relevant articles was retrieved from PubMed for the period between inception and 14 August 2023. There were no restrictions on article type, study design, or language. All titles and abstracts were screened by at least one author or assistant, yielding 2302 articles for further review. Additional articles not generated by the initial search query were included if deemed pertinent upon a non-exhaustive review of cited references. The body of relevant articles was then used as the foundation for developing each section of this narrative review, although it was not required that all articles be incorporated into the manuscript.

## 5. Role of Prebiotics and Probiotics for Various Gastrointestinal Conditions

### 5.1. Diarrhea

Probiotics have been shown to confer benefits in the prevention and treatment of some types of diarrhea, as described below; however, little to no information is available regarding the role of prebiotics for diarrhea.

#### 5.1.1. Infectious Diarrhea

One systematic review and meta-analysis found that *Saccharomyces boulardii* CNCM I-745 significantly decreased the incidence of traveler’s diarrhea [48]. A separate meta-analysis by Fagnant et al. found that prebiotics and probiotics modestly reduced the risk of GI tract infection in adults; however, the study was limited by a high risk of bias and heterogeneous interventions and could not assess the effects of specific prebiotic or probiotic strains [49].

For the treatment of infectious diarrhea, a 2021 meta-analysis showed that multiple single-strain and multi-strain probiotics significantly reduced the duration of acute diarrhea in children; *Saccharomyces boulardii* was the most effective probiotic strain overall; however, *Limosilactobacillus reuteri*, *Bifidobacterium lactis*, *Lactobacillus* species (spp.) plus *Bifidobacterium* spp. plus *Saccharomyces* spp., and *Bacillus* spp. plus *Enterococcus* spp. plus *Clostridium* spp. were also shown to be effective [50]. Multiple other meta-analyses confirmed that probiotics could decrease the duration of diarrhea and length of hospitalization, with *Saccharomyces* and *Bifidobacterium* frequently cited to be more effective than *Lactobacillus* [51,52]. Importantly, the dose and timing of probiotic administration seemed to impact their effect. Two meta-analyses demonstrated that higher doses of *Lacticaseibacillus rhamnosus* GG (≥10^10^ colony-forming units [CFU]) were more effective in reducing the duration of diarrhea [53,54]. In contrast, a previous systematic review and meta-analysis by the Cochrane Collaboration in 2020 found no difference in the incidence or duration of acute infectious diarrhea in the probiotic versus control groups [55].

The effect of probiotics on infectious diarrhea in children may also be influenced by socioeconomic status. One meta-analysis found that the combination of *Saccharomyces boulardii* and zinc reduced the duration of acute diarrhea in children in low- and middle-income countries [56]; this benefit was not seen in another meta-analysis examining the duration of diarrhea in children in developed countries receiving probiotic supplementation [57].

Overall, there is convincing evidence that certain probiotic strains can be safe and effective in preventing and treating infectious diarrhea. Available studies suggest that *Saccharomyces boulardii* may be relatively more efficacious; however, further research is needed to identify the most optimal strain(s) and dosage of probiotics as well as the role of prebiotics for acute diarrhea [58].

#### 5.1.2. Antibiotic-Associated Diarrhea (AAD)

Probiotics have been associated with a significant reduction in AAD without a significant increase in adverse effects [59,60]. *Saccharomyces boulardii*, *Lactocaseibacillus rhamnosus* GG, and probiotic mixtures were found to be particularly effective in one meta-analysis [61]. Another randomized controlled trial (RCT) demonstrated that a probiotic drink containing *Lactobacillus casei* DN 114001 was effective in preventing AAD [62]. Like infectious diarrhea, the dose and timing of probiotic administration impact their effect on antibiotic-associated diarrhea. Probiotics were demonstrated to be particularly effective at higher doses (≥5 billion CFUs/day) and when administered within two days of antibiotic treatment for elderly adults [63,64]. No RCTs have examined the effect of prebiotics on the prevention or treatment of antibiotic-associated diarrhea.

#### 5.1.3. *Clostridioides difficile* Infection (CDI)

*Saccharomyces boulardii* has been shown to produce a protease that inhibits *C. difficile* toxins A and B, which may underlie its potential benefit in CDI [65]. For hospitalized patients receiving antibiotics, a 2017 meta-analysis demonstrated that administration of probiotics closer to the first dose of antibiotics reduced the risk of CDI by >50%, with no increased risk for adverse effects. [66]. However, the protective effect of probiotics was only seen in patients with a >5% baseline risk of *Clostridioides difficile*-associated diarrhea (CDAD) in another meta-analysis [67]. With low quality of evidence, the American Gastroenterological Association (AGA) recommends using one of the following single- or multi-strain probiotic formulations for prevention of CDI in patients receiving antibiotics: (1) *S. boulardii*, (2) *L. acidophilus* CL1285 plus *L. casei* LBC80R, (3) *L. acidophilus* plus *L. delbrueckii* subsp. *bulgaricus* plus *Bifidobacterium bifidum*, or (4) *L. acidophilus* plus *L. delbrueckii* subsp. *bulgaricus*, *B. bifidum*, and *Streptococcus salivarius* subsp. *thermophilus* [68]. Additionally, a recent phase 2 clinical trial demonstrated that high-dose VE303, a combination of eight strains of commensal *Clostridia*, was effective in preventing recurrent CDI in at-risk patients [69].

#### 5.1.4. Chemotherapy- and Radiation-Induced Diarrhea

Chemotherapy and radiation therapy can cause intestinal mucositis and diarrhea by increasing intestinal permeability via intestinal crypt apoptosis and villous atrophy and by reducing the diversity of the gut microbiota (i.e., reduced levels of *Bifidobacterium*) [70]. The mechanism by which this gut dysbiosis occurs is not entirely clear, but some chemotherapy agents such as etoposide have demonstrated direct antibacterial activity, especially against Gram-positive bacteria [71]. Thus, probiotics could be a useful adjunct to traditional therapies for chemotherapy- and radiation-induced diarrhea; however, existing data are limited. A review by the Cochrane Collaboration did not find high-quality evidence showing a significant association between probiotics and chemotherapy- or radiation-induced diarrhea; however, no adverse effects were seen [72].

One Japanese RCT examining abemaciclib-induced diarrhea in patients with breast cancer found that supplementation with *Bifidobacterium* with or without trimebutine maleate did not decrease the incidence of grade 2 or greater diarrhea [73]. Another meta-analysis found that probiotics can prevent and treat chemotherapy-induced diarrhea without significant adverse effects, though the results were limited by heterogeneity and poor methodological quality amongst the included trials. Most included studies examined probiotics containing *Bifidobacterium* spp. and/or *Lactobacillus* spp., but no further strain-specific effects were reported [74].

For radiation-induced diarrhea, a meta-analysis in 2017 found that probiotics significantly reduced the incidence of radiation-induced diarrhea in patients with abdominal or pelvic cancers. The probiotic strains found to have an effect were *Lactobacillus acidophilus* plus *Bifidobacterium bifidum*, *L. acidophilus* LAC-361 plus *B. longum* BB-536, and VSL#3^®^ (a multi-strain probiotic including *L. paracasei*, *L. plantarum*, *L. acidophilus*, *L. helveticus*, *B. longum*, *B. breve*, *B. infantis*, and *S. thermophilus*). A synbiotic containing *L. acidophilus* and lactulose was also found to be effective in reducing radiation-associated diarrhea [75,76]. Studies examining prebiotics were limited; however, one small RCT found that supplementation with resistant starch did not reduce the incidence of radiation-induced proctitis [77].

Taken together, there is a signal towards a benefit of probiotics for radiation-associated diarrhea, but there is no convincing evidence for the use of probiotics for chemotherapy-induced diarrhea.

### 5.2. Constipation

There are observed differences in the gut microbiome of patients with constipation, namely decreased levels of *Bifidobacteria* and *Lactobacilli* and increased levels of *Bacteroides* [78,79,80]. Prebiotics and probiotics are therefore thought to be helpful in constipation by increasing levels of *Bifidobacteria* and *Lactobacilli*; the resultant production of SCFAs may regulate motility by increasing the release of serotonin and stimulating enteric or vagal nerves acting on colonic smooth muscle [81,82]. Studies have examined the effect of different prebiotic types and probiotic species on various aspects of constipation including stool frequency, stool consistency, defecation pain, overall response to treatment, and quality of life, lending to significant heterogeneity between studies in pooled analyses.

#### 5.2.1. Prebiotics for Constipation

One RCT demonstrated that the consumption of 15 g of chicory inulin daily for 28 days by elderly adults with constipation led to increased levels of *Bifidobacterium* and an improvement in constipation and quality of life [83]. Some gastrointestinal side effects, such as increased flatulence, were noted with inulin supplementation but did not lead to discontinuation in the study. Two additional RCTs demonstrated that inulin consumption led to significantly increased stool frequency in constipated adults [84,85].

In addition to inulin, Deshipu stachyose granules (DSGs), a mixture of alpha-galacto-oligosaccharides, have also shown efficacy in constipation. An RCT from 2017 demonstrated that treatment with 5 g per day of DSGs for 14 days led to increased levels of fecal *Bifidobacteria* and *Lactobacilli* and decreased levels of fecal *Clostridium perfringes* in healthy patients. Furthermore, treatment with 5 g of DSGs daily for 30 days in constipated patients led to an improvement in defecation ease and frequency, as well as softer stools; no adverse effects were noted [86]. Similarly, daily consumption of 11 g of GOSs significantly increased stool frequency in adults with ≤3 bowel movements per week in another RCT. The authors also found a dose–response relationship between GOSs and levels of fecal *Bifidobacterium* [87]. A 2015 meta-analysis confirmed that GOSs significantly increased stool frequency with no heterogeneity between studies. However, this benefit was not seen with inulin, though there was significant heterogeneity noted. There were no reported adverse effects [88].

In summary, the existing evidence suggests that prebiotics, especially GOSs, are safe and effective in improving constipation and may exert their effects by modulating the gut microbiome and *Bifidobacterium* levels, in particular.

#### 5.2.2. Probiotics for Constipation

A systematic review and meta-analysis found that probiotics, specifically *Bifidobacterium lactis*, significantly increased stool frequency in adults with chronic constipation. This effect on stool frequency was not seen with other probiotic strains, such as *Bacillus coagulans* Unique IS-2 or *Lacticaseibacillus casei* Shirota, or with mixtures of probiotics; however, *B. coagulans* Unique IS-2 was noted to improve abdominal pain and defecation pain. Overall, probiotics were also found to improve response to treatment and integrative symptom scores, but no species- or strain-specific effects were identified for these metrics. Only minor adverse effects were reported such as loose stools, bloating, and abdominal discomfort, which were not significantly different between the probiotic and control groups [89,90]. The RCT by Yoon et al., which was included in the prior meta-analysis, found that daily supplementation with *Streptococcus thermophilus* MG510 (3 × 10^8^ CFU) and *Lactiplantibacillus plantarum* LRCC5193 (1 × 10^8^ CFU) significantly improved stool consistency in adults with constipation after 4 weeks of treatment. Interestingly, the relative abundance of *Lactiplantibacillus plantarum* in the fecal microbiome persisted in the probiotic group four weeks after discontinuation of the supplement, suggesting that it may confer a prolonged benefit [91].

A Brazilian RCT by Mitelmão et al. compared the efficacy of a probiotic mixture containing three strains of *Lactobacillus* and *Bifidobacterium*, another mixture containing eight strains, and conventional fiber therapy in adults with constipation. All interventions were safe and effective in improving symptoms of constipation; however, no significant difference was seen between the groups [92].

A separate meta-analysis found that multispecies probiotics significantly improved defecation frequency and fecal incontinence in children with chronic constipation, but there was no significant effect on treatment success, abdominal pain, and painful defecation [93]. *Limosilactobacillus reuteri* was studied in five of the included RCTs, but a subgroup analysis could not be performed due to inconsistent outcome reporting. *L. rhamnosus*, *B. longum*, and *Saccharomyces boulardii* were also included in the systematic review, in addition to several multispecies probiotics, which were found to have a more significant benefit than probiotics with a single species.

Duration of probiotic treatment and concomitant laxative use may also impact its efficacy. An RCT by Šola et al. found that a liquid probiotic formulation containing *Bifidobacterium animalis* susp. *Lactis* BLC1, *Lactobacillus acidophilus* LA3, and *Lactobacillus casei* BGP93 significantly increased the cumulative number of bowel movements after the 10th week of treatment in elderly patients without concomitant laxative use. No adverse events were noted in the treatment group and no significant differences were seen in the safety-monitoring labs [94].

Taken together, these results are promising that probiotics, especially multi-strain formulations, are likely safe and effective in alleviating some aspects of constipation; however, more studies are needed to determine the optimal strain(s), dose, and duration of treatment to inform clinical recommendations. Additionally, more studies comparing probiotics to conventional fiber therapy are needed to demonstrate superiority given the additional considerations of cost, stability, and storage associated with probiotics.

#### 5.2.3. Synbiotics for Constipation

Data were more limited for synbiotics and results were inconclusive. An RCT by Baştürk et al. found that a synbiotic containing *L. casei*, *L. rhamnosus*, *L. plantarum*, *B. lactis*, fiber, polydextrose, FOSs, and GOSs significantly improved stool frequency and symptoms of constipation in children after 4 weeks of treatment [95]. In contrast, a 2022 meta-analysis found that synbiotics did not significantly improve stool output or integrative symptom scores in constipated adults. Four studies were included in the pooled analysis and the synbiotics tested were (1) *Lactiplantibacillus plantarum* LP01 and *Bifidobacterium lactis* BB12 plus inulin and oligofructose, (2) *Lacticaseibacillus casei* CRL431 and *B. lactis BB12* plus inulin and oligofructose, (3) *B. lactis* LMG P-28149 and FOS, and (4) *L. paracasei* Lpc-37, *L. rhamnosus* HN001, *L. acidophilus* NCFM, and *B. lactis* HN019 plus FOS [89].

### 5.3. Irritable Bowel Syndrome (IBS) and Disorders of Gut–Brain Interaction (DGBIs)

Per the Rome IV criteria, irritable bowel syndrome (IBS) is diagnosed in patients with recurrent abdominal pain at least once weekly, on average, over the prior three months that is associated with at least two of the following symptoms: pain related to defecation, change in stool frequency, and change in stool form or appearance. It can be further classified as constipation-predominant IBS (IBS-C), diarrhea-predominant IBS (IBS-D), IBS with mixed bowel habits (IBS-M), or IBS unclassified (IBS-U) [96]. The pathophysiology is multifactorial and not fully understood, but is thought to involve motility dysfunction, alterations in the gut microbiota and enteric nervous system, and low-grade inflammation—all of which may be regulated by prebiotics and probiotics.

#### 5.3.1. Prebiotics for IBS

The role of prebiotics for IBS is complex and not well understood, as some prebiotic types may be beneficial and others may cause harm [97,98]. This is unsurprising, as many patients with IBS are sensitive to FODMAPs (Fermentable Oligosaccharides, Disaccharides, Monosaccharides, Additionally, Polyols), which include FOSs and GOSs [98]. Three RCTs demonstrated that food products or beverages containing inulin led to improvement in stool parameters in patients with IBS-C [99,100,101]. One of these studies, which tested a functional drink containing inulin, menthol, and pyridoxine, found increased heartburn in the treatment arm, but otherwise, no major adverse effects were noted [100]. In contrast, oligofructose and FOSs were not effective in reducing IBS symptoms; in fact, FOS supplementation led to worsening of symptoms after 4–6 weeks in one study, though there was no difference between the intervention and placebo groups at 12 weeks [102,103]. Another RCT demonstrated that GOS was effective in reducing global IBS symptom scores at doses of 3.5 g/day and 7 g/day; however, the group receiving the lower dose experienced fewer side effects [104]. Though not statistically significant, one study found that short-chain FOSs (scFOSs) tended to reduce rectal sensitivity in patients with IBS-C. scFOSs also significantly reduced anxiety scores and increased fecal *Bifidobacteria* compared to placebo [105]. A 2019 systematic review and meta-analysis concluded that prebiotics did not significantly impact symptom scores in patients with IBS, though interpretation was limited by study heterogeneity. The study also showed that prebiotics increased the relative abundance of *Bifidobacteria* in patients with IBS, with subgroup analysis highlighting that inulin-type fructans and doses of prebiotics > 6 g/day increased levels of *Bifidobacteria* [106].

In summary, inulin and GOSs may be helpful for IBS, though additional studies are needed to confirm their benefit, and adverse effects may be associated with higher doses. Evidence for FOSs was inconsistent and suggested a possible deleterious effect of FOSs on IBS symptoms.

#### 5.3.2. Probiotics for IBS

A systematic review and meta-analysis from 2018 found that probiotics significantly reduced the risk of persistent symptoms compared to placebo in patients with IBS. The study identified that the combination probiotic, LacClean Gold (contains *Bifidobacterium longum*, *B. bifidum*, *B. lactis*, *Lactobacillus acidophilus*, *Lacticaseibacillus rhamnosus*, and *Streptococcus thermophilus*), and a seven-strain combination of three *Bifidobacterium* strains, three *Lactobacillus* strains, and one *Streptococcus* strain showed a significant benefit over placebo. Additionally, the individual strains *Lactiplantibacillus plantarum* DSM 9843, *Escherichia coli* DSM 17252, and *Streptococcus faecium* were superior in reducing the risk of persistent symptoms. While there was a trend towards benefit for *Bifidobacterium* (*p* = 0.05), no significant strain-specific effects were identified for global IBS and abdominal pain scores. Pooled analysis of combinations of probiotics demonstrated a benefit for this outcome, with VSL#3^®^ showing a trend towards benefit [107]. Another small RCT demonstrated that a combination probiotic, Bifiform (containing *Enterococcus faecium* and *Bifidobacterium longum)*, was more effective in treating post-infectious IBS compared to standard complex therapy alone, which included an antispasmodic drug, an antibiotic, and a drug to normalize the consistency of feces [108].

Overall, probiotics appear to improve IBS symptoms, with certain single-strain and combination-strain probiotics emerging as superior.

#### 5.3.3. Synbiotics for IBS

A 2021 review article summarized the results of 10 clinical studies examining different synbiotic formulations in IBS [97]. Most studies demonstrated a significant improvement in at least one IBS marker, from abdominal bloating and pain to SCFA levels. More studies are needed to confirm the benefit of each synbiotic formulation and better define their clinical impact.

### 5.4. Small Intestinal Bacterial Overgrowth (SIBO)

SIBO is characterized by an increase in the number of bacteria in the small bowel in a distribution more commonly associated with the colon, leading to gastrointestinal symptoms and malabsorption. Antibiotics are the mainstay of therapy, however, recurrence of SIBO is extremely common, often necessitating re-treatment which can increase the risk of antibiotic resistance, diarrhea, and food intolerances [109]. As a result, there is interest in harnessing probiotics in the treatment of SIBO, given their ability to produce antimicrobial substances, compete with pathogenic microbes for nutrients and adhesion to the gastrointestinal mucosa, increase motility, and help restore balance in the gut microbiota after antibiotic therapy. Unfortunately, research on prebiotics and probiotics for SIBO is scarce, underlining the need for additional research to confirm their clinical safety and efficacy.

#### 5.4.1. Prebiotics for SIBO

Only one RCT examined the role of prebiotic supplementation for SIBO. Rosania et al. showed that treatment with rifaximin for seven days followed by FOSs for seven days led to a significant improvement in four out of six symptoms evaluated (diffuse abdominal pain, left iliac pain, meteorism, and flatulence) in patients with SIBO [110].

#### 5.4.2. Probiotics for SIBO

A 2017 systematic review and meta-analysis found that probiotics led to higher rates of SIBO decontamination compared to placebo and metronidazole; probiotics plus antibiotics were more effective than probiotics alone. Additionally, there was a significant decrease in H_2_ levels detected on hydrogen breath testing after taking probiotics and an improvement in abdominal pain scores, but there was no significant impact on daily stool frequency. Probiotics were not found to have a significant effect on SIBO incidence in the pooled analysis [111].

Among patients with SIBO in the setting of systemic sclerosis, one RCT showed that treatment with *Saccharomyces boulardii* with or without metronidazole led to a significant improvement in pain and bloating compared to metronidazole alone, with no serious adverse effects [112]. Notably, one RCT demonstrated that supplementation with the Bifidobacterium triple-viable capsule (contains *B. longum*, *L. acidophilus*, and *Enterococcus faecalis*) significantly improved symptoms and rates of SIBO resolution compared to placebo in patients with SIBO and GI malignancies [113,114]. An RCT by Rosania et al. showed that rifaximin followed by *Lacticaseibacillus casei* improved symptoms of diffuse abdominal pain, left iliac pain, meteorism, flatulence, and nausea in patients with SIBO. Rifaximin followed by *Lacticaseibacillus casei* was found to be more effective in improving symptoms than rifaximin followed by FOSs, though this difference was not statistically significant [110].

Available evidence suggests that probiotics, especially *L. casei*, can improve symptoms associated with SIBO and may exhibit a synergistic effect when used with antibiotics for SIBO treatment.

#### 5.4.3. Synbiotics for SIBO

A small RCT demonstrated that the addition of a synbiotic containing *Bacillus coagulans* and FOSs to maintenance antibiotic therapy led to a significant improvement in abdominal pain and gastrointestinal symptoms, such as flatulence, belching, and diarrhea, compared to antibiotics alone. A greater proportion of patients in the probiotic group had a negative hydrogen breath six months after treatment, though this difference was not statistically significant [115].

### 5.5. Inflammatory Bowel Disease (IBD)

The role of specific gut microbial communities in defining the metabolomic products of dietary intake, which can in turn exert pro- or anti-inflammatory effects, has motivated the exploration of microbiome manipulation approaches for IBD [116]. The conversion of some prebiotics by microbial fermentation into SCFAs provides a mechanistic basis for their anti-inflammatory effects. SCFAs modulate inflammation through the induction of regulatory T-cells in the colon, partial suppression of macrophage activation, and inhibition of nuclear factor kappa-light-chain-enhancer of activated B (NF-κB) activation in lamina propriate macrophages [117,118,119]. SCFAs can also regulate intestinal epithelial barrier function, thus reducing bacterial translocation and mucosal antigen presentation [120].

#### 5.5.1. Prebiotics for IBD

Given the potential immunological benefits, several RCTs and an open-label observational trial investigated the role of prebiotics for the induction and maintenance of remission in IBD.

For ulcerative colitis (UC), a Japanese RCT with 40 participants found the FOS 1-kestose at 10 g/day to be superior to a maltose placebo for the induction of clinical remission [121]. Three RCTs did not otherwise find a difference between oligofructose-enriched inulin (OF-IN) at 10–12 g/day with their respective controls (OF-IN at 7.5 g/day, maltodextrin placebo, no intervention) for the induction of clinical remission [122,123,124]. There was nonetheless a reduction in fecal calprotectin concentrations observed in two of the RCTs with OF-IN at 12–15 g/day but not with the lower-dose OF-IN at 7.5 g/day or maltodextrin placebo [122,123]. As for other prebiotics, two RCTs with 59 participants found that germinated barley foodstuff (GBF) at 20–30 g/day for 2–4 weeks decreased gastrointestinal symptoms [125,126]. In an RCT with 51 participants with active UC, roasted *Plantago ovata* seeds at 3.6 g/day led to less abdominal tenderness than the roasted wheat flour control by week 8 [127]. More granular data on rates of remission comparing both prebiotics and their respective controls were not reported.

For maintenance of remission in UC, an open-label observational trial with 59 participants found GBF at 20 g/day to be associated with lower clinical activity scores and cumulative relapse rates than the no-intervention control by week 52 [128]. RCTs did not otherwise find relapse rates for those who received OF-IN, oat bran, or *Plantago ovata* seeds to differ from their respective controls [129,130,131,132].

For Crohn’s disease (CD), the data on prebiotics are even more sparse. Two RCTs evaluated OF-IN at 15–20 g/day and found no benefit for the induction of clinical remission [133,134]. There are no controlled trials evaluating the role of prebiotics in the prevention of clinical relapse in CD.

While prebiotics are generally considered safe, pooled analyses indicate that OF-IN may lead to an increased risk of adverse events compared to their respective controls [122,123,133,134]. The complaints included bloating and flatulence, which are not surprising from the consumption of oligosaccharides. There were otherwise no serious adverse effects. As for 1-kestose, lactulose, GBF, *Plantago* seeds, and psyllium, adverse event rates were no different than those of controls in RCTs.

Due to the very small sample sizes and some risk of bias among these studies of prebiotics for IBD, the certainty of evidence is generally very low and no conclusions can be made about the efficacy of prebiotic supplementation for IBD at this time. There are nonetheless some data to support the use of plant-based diets, which are rich in dietary fibers, for reductions in symptoms and, potentially, inflammation [135,136]. Fruits and vegetables are also a consistently important component of anti-inflammatory diets found to be helpful for IBD. Care should nonetheless be exercised when recommending fiber for patients with stricturing CD [137].

#### 5.5.2. Probiotics for IBD

Unlike with prebiotics, there are more data on the use of probiotics for IBD, although there is still much need for additional investigation. In a systematic review and meta-analysis by the Cochrane Collaboration, probiotics were overall effective for the induction of clinical remission in UC [138]. Probiotics were also effective for achieving clinical improvement, endoscopic improvement, and histologic improvement, but not histologic remission. However, in subgroup analysis, single-strain probiotics (including *E. coli* Nissle 1917, *L. reuteri* ATCC 55730, *B. longum*, and *L. casei*) were not effective individually or in aggregate for the induction of clinical remission [139,140,141,142]. By contrast, two RCTs with 29 and 147 participants, respectively, found VSL#3^®^ at 450–3600 × 10^9^ CFU/day to be effective for the induction of clinical remission in UC [143,144]. Another RCT with 144 participants also found VSL#3^®^ at 3600 × 10^9^ CFU/day to improve disease activity scores and rectal bleeding, although the differences were not significantly different from placebo [145]. There were no overall differences in minor or serious adverse events when comparing probiotics with placebo [138]. In a separate systematic review and meta-analysis by the Cochrane Collaboration, three RCTs of three different multi-strain probiotics did not identify any that were effective individually or in aggregate for the maintenance of remission in UC [146]. There is no RCT that specifically examined the efficacy of VSL#3^®^ for the prevention of clinical relapse in patients who were in remission at baseline; however, an RCT that included a longitudinal follow-up after initial randomization to evaluate the induction of remission found that those assigned to the VSL#3^®^ arm had lower rates of relapse within 1 year (21.4% vs. 73.3%; *p* = 0.014) [143].

For patients who underwent ileal pouch anal anastomosis and subsequently developed pouchitis, there are some RCTs that demonstrated the benefit of probiotics for primary prevention [147,148]. The AGA, however, graded the available evidence to have very low certainty and thus has not provided a specific recommendation for this [149]. One RCT with 20 participants of a single-strain probiotic with *L. rhamnosus* GG did not find a benefit for the treatment of pouchitis [150]. A pooled analysis with two observational trials nonetheless found a benefit of probiotics for clinical response, albeit with very low certainty of evidence [149]. In this scenario, the AGA similarly has no formal recommendation. On the other hand, guidelines from the AGA suggest consideration of probiotics for the prevention of recurrent pouchitis [149]. This conditional recommendation was based on three RCTs that evaluated the efficacy of VSL#3^®^ for recurrent pouchitis [151,152,153].

Similar to prebiotics, evidence for probiotic use in CD is sparse and appears less promising. In a systematic review and meta-analysis by the Cochrane Collaboration on probiotics for induction of remission in CD, only two RCTs with 46 participants met the inclusion criteria [154]. One RCT with 11 participants evaluated a single-strain probiotic with *L. rhamnosus* GG [155], while the other RCT with 35 participants evaluated a synbiotic with *B. longum* and OF-IN [156]. The very small sample sizes and use of single-strain probiotics indicate that the data are currently far too scarce to yet make any conclusions about the efficacy—or lack thereof—of probiotics for CD.

#### 5.5.3. Synbiotics for IBD

Given the theoretical and demonstrated benefits of prebiotics and probiotics, respectively, the synergistic benefit of synbiotics has also been investigated. In one RCT with 94 participants that compared psyllium at 8 g/day, a probiotic with *Bifidobacterium longum* at 2 × 10^9^ CFU/day, and their synbiotic for 4 weeks, CRP and quality-of-life measures improved from baseline among those who received the synbiotics, but not among those in the other treatment arms [157].

### 5.6. Celiac Disease

Patients with celiac disease may experience ongoing gastrointestinal symptoms despite following a strict gluten-free diet. Therefore, researchers have investigated the use of probiotics as an adjuvant treatment for celiac disease. In 2020, Seiler et al. completed a systematic review and meta-analysis and found that supplementation with *B. infantis* or a probiotic strain containing *L. casei*, *L. plantarum*, *B. lactis*, *B. breve* Bbr8, and *B. breve* B110 improved GI symptoms when assessed by the GI Symptoms Rating Scale (*p* = 0.0002) including abdominal distention, bloating, constipation, vomiting, and diarrhea [158,159,160,161]. The authors reported the quality of evidence was low for the effect of probiotics on overall gastrointestinal symptoms with a high risk of bias. *Bifidobacteria* levels were increased after the use of probiotics in two meta-analyses [158,162]. Another systematic review reported that a gluten-free diet in children with celiac disease supplemented by probiotic therapy can alter fecal microbiota to typical conditions of healthy individuals and reduce serum pro-inflammatory cytokines [163]. It should be noted that there are limited available prospective studies conducted in North America, Asia, or Africa, thus reducing the generalizability of the available literature. At this time, considering the limited body of evidence available, a recommendation cannot be made on the use of probiotics in adult and pediatric patients with celiac disease as a complementary therapy to a strict gluten-free diet. High-quality, prospective clinical trials, including large RCTs, are greatly needed to better explore the use of probiotics in individuals with celiac disease.

### 5.7. Helicobacter pylori Infection

*Helicobacter pylori* is a very common infection globally and increases the risk of developing peptic ulcers or gastric cancer if not properly eradicated [164]. Every effort should be made to address factors that might contribute to eradication failure as the odds of successful eradication decrease with each failed treatment attempt. Several guidelines exist to guide clinicians in selecting the recommended intervention for eradication as well as management after failed treatments. The use of probiotics has been studied in the evaluation and treatment of *H. pylori* infection due to their potential to promote intestinal health and immunity. In vitro studies, animal studies, and clinical observations have reported that probiotics may reduce side effects in combination with traditional *H. pylori* therapies. It is suggested that probiotics may directly compete with *H. pylori* to help restore the intestinal microbial environment, increasing IgA production and strengthening the mucosal barrier against pathogens [165].

A prospective study including 167 patients diagnosed with *H. pylori* infection found that treatment with triple-eradication therapy and probiotic cultures (*Lactobacillus acidophilus* Rosell-52, *Lactobacillus acidophilus* Rosell-11, *Bifidobacterium infantis* Rosell-1755 and *Saccharomyces boulardii*) was more successful in achieving eradication success compared with triple-eradication therapy alone (*p* < 0.05). There was no significant difference in the incidence of adverse events among both groups [166]. Additionally, 199 patients with confirmed *H. pylori* infection treated with standard sequential therapy (omeprazole plus amoxicillin for 5 days followed by omeprazole, clarithromycin, and metronidazole for five days) and *Saccharomyces boulardii* had higher eradication rates (*p* = 0.02) and a significantly lower overall incidence of adverse events (*p* < 0.001) compared to sequential therapy alone [167].

A meta-analysis including eight RCTs suggested that supplementation of *Lactobacilli* may be effective in increasing eradication rates during the initial treatment of *H. pylori* with a positive impact on some therapy-related side effects [168]. Additionally, eight tertiary hospitals in a prospective placebo-controlled study found that four probiotic strains (*Lactobacillus acidophilus*, *Lactiplantibacillus plantarum*, *Bifidobacterium lactis*, and *Saccharomyces boulardii*) increased eradication rates (92.0% vs. 86.8%; *p* = 0.028) and decreased side effects of patients (17.0% vs. 50.7%; *p* < 0.00001) compared with individuals who did not receive probiotic supplementation [169]. A different probiotic therapy of four strains (*Lacticaseibacillus rhamnosus* GG, *L. rhamnosus*, *Bifidobacterium breve,* and *Propionibacterium freudenreichii*) versus placebo in 47 subjects with *H. pylori* infection did not find a significant change in eradication rate (*p* = 0.42) but noted less treatment-related symptoms (*p* = 0.038) as measured by the total symptom score change [170]. Furthermore, supplementation of a synbiotic yogurt containing pectin, GOSs, *L. acidophilus* LA-5, *B. lactis* BB-12, *L. bulgaricus*, and *S. thermophilus* was found to suppress *H. pylori* infection in 59 adults [171].

At this time, the AGA does not have a formal recommendation on the use of probiotics or prebiotics for the treatment of *H. pylori*. Probiotics may become a future treatment when used alone or in combination with best-practice treatment against *H. pylori* infection. However, the many different strains, formulas, doses, and timing of probiotics available and researched as adjuncts against *H. pylori* make it difficult to standardize the results and make a definitive recommendation at this time; thus, their use should be considered experimental.

### 5.8. Colon Cancer Prevention

Colon cancer remains one of the most common cancer types worldwide, and evidence-based practice guidelines consistently describe the link between diet, lifestyle, and aging and its development. An increase in gastrointestinal mucosal permeability and subsequent inflammation are believed to play a role in the pathophysiology of gastrointestinal cancers including colorectal cancers. Therefore, recent research efforts have focused on exploring interventions that may aid in the prevention of developing colon cancer. The intestinal microbiota is widely considered for its role in maintaining balanced homeostasis and immunomodulation and is more recently being investigated for its potential antitumor properties. Specifically, lactic acid-producing bacteria have been shown to play a role in the regression of carcinogenesis, highlighting the interaction between epithelial, immune, and bacterial metabolites [172]. An increased abundance of *Escherichia coli*, *Enterococcus faecalis*, *Fusobacterium nucleatum*, *Bacteroides fragilis*, and *Streptococcus gallolyticus* and a decreased abundance of *Clostridium*, *Roseburia*, *Faecalibacterium*, and *Bifidobacterium* have been observed in patients diagnosed with colon cancer [173]. Several in vivo, in vitro, and clinical studies have reported that probiotics may prevent the development of colon cancer [174]. The mechanism of action of probiotics on carcinogenesis, mainly regarding the use of *Lactobacillus* and *Bifidobacterium*, has not been fully elucidated as their effects are diverse and complex. In addition, there are animal studies demonstrating that dietary intake of inulin prevents preneoplastic changes and inflammation, which promote colon cancer development [175]. In contrast, no reduction in colon cancer risk was seen among patients who received supplementation with oligofructose-enriched inulin for 6 months in a phase 2 clinical trial [176].

At present, there are limited studies with sufficient follow-up results and reproducibility investigating the use of prebiotics and probiotics for cancer biotherapy. Further studies reporting on probiotics in the field of oncology are greatly needed to explore the potential in identifying bacterial species and strains with anti-cancer properties in the fight against the development of cancer.

## 6. Safety and Adverse Effects

While many studies have demonstrated the potential benefits of prebiotics and probiotics, data regarding their safety and adverse effects are limited. Adverse effects are often poorly reported in existing studies, and many often focus on short-term gastrointestinal side effects without monitoring for certain infections or other longer-term effects. Prebiotics are generally considered to be safe, but a dose–response relationship exists for adverse effects, primarily diarrhea, bloating, and flatulence, owing to their osmotic properties [8,177]. Similarly, probiotics appear to be safe in average-risk patients, with a 2011 meta-analysis reporting no significant increase in the risk of overall adverse events, including serious adverse effects, in patients receiving short-term probiotic supplementation [178]. Probiotics have, however, been shown to have rare but serious consequences in vulnerable populations, such as preterm infants and elderly, critically ill, post-surgical, and immunocompromised patients. A 2014 systematic review described several cases of bacteremia with *Lactobacillus* strains (*L. rhamnosus* GG, in particular) and fungemia in ICU patients with a central venous catheter receiving *S. boulardii* [179]. Another study examined the efficacy of a synbiotic (composed of *Bifidobacterium*, *Lactobacillus*, corn starch, and maltodextrins) for preventing infections in patients with severe acute pancreatitis; the results showed a 2.5-fold higher mortality rate (95% confidence interval: 1.2–5.3) and incidence of bowel ischemia in the treatment arm compared to placebo [180]. In October of 2023, the FDA released a warning advising against the use of probiotics in preterm infants due to cases of fatal sepsis. Researchers have been investigating whether heat-killed or UV-inactivated probiotic strains may be safer for the host while still exerting their anti-inflammatory effects [181]. Overall, prebiotics and probiotics are likely safe in most individuals, but no definite recommendation can be made given limited safety data and significant variability in prebiotic type, probiotic strain, doses used, and outcome reporting in existing studies.

## 7. Conclusions

Prebiotics and probiotics are known to alter the composition and function of the gut microbiota, allowing them to exert local and systemic effects through the action of molecules such as SCFAs. Prebiotics, such as inulin and GOSs, have demonstrated efficacy in the treatment of constipation in several studies. Results were mixed for IBS, and certain prebiotic types were shown to exacerbate symptoms of IBS. Data were sparse for IBD and SIBO, and no conclusions could be drawn. There were very few studies examining the utility of prebiotics for celiac disease, *H. pylori* infection, and colon cancer prevention. Higher doses were associated with more gastrointestinal side effects, such as bloating and flatulence, but prebiotics were well tolerated overall. There were far more data for probiotics, with *Lactobacilli* and *Bifidobacterium*, as well as the yeast *Saccharomyces*, being among the most-studied species for GI disorders. Available studies supported the benefit of probiotics for infectious diarrhea, antibiotic-associated diarrhea, and constipation. Probiotics demonstrated a synergistic effect when used with antibiotics for SIBO and *H. pylori* infection. The strongest signal for probiotic use in IBD was for the prevention of recurrent pouchitis. As they are live microorganisms, probiotic use raises the additional considerations of cost, stability, and safety, particularly for high-risk populations.

In summary, prebiotics and probiotics demonstrate promise in the prevention and treatment of certain GI disorders, as an adjunct or alternative to conventional therapies. However, these data are difficult to translate to specific clinical guidelines given the wide variation in prebiotic type(s), probiotic strain(s), dose, and/or duration of treatment used in each study. Furthermore, standardized reporting of safety outcomes and studies examining their potential long-term effects are severely lacking. For each gastrointestinal indication, additional large-scale, high-quality, and strain-specific RCTs are needed to validate the safety and efficacy of prebiotics and probiotics seen in these smaller RCTs, and make recommendations for the general public.

## Supplementary Materials

The following supporting information can be downloaded at https://www.mdpi.com/article/10.3390/nu16060778/s1, Supplemental Material 1: Literature Search Query; Figure S1: Pre-probiotics microbiome; Table S1: Summary of prebiotics, probiotics, and synbiotics shown to be effective for GI conditions.
