# Supplementary material for: Prebiotics and Probiotics for Gastrointestinal Disorders
Source: Nutrients. 2024 Mar 9;16(6):778. doi: 10.3390/nu16060778 (PMC10975713; doi:10.3390/nu16060778)
Supplement: Supplementary file 1 [file nutrients-16-00778-s001.zip › Prebiotics and Probiotics_Table S1 FINAL 3-8-24.pdf]

Table S1. Summary of prebiotics, probiotics, and synbiotics shown to be effective for gastrointestinal (GI) conditions*

		Infectious diarrhea	Antibiotic-associated diarrhea	Clostridioides difficile infection	Radiation-induced diarrhea	Constipation	Irritable Bowel Syndrome	Small Intestinal Bacterial Overgrowth	Ulcerative Colitis	Pouchitis	Celiac Disease	Helicobacter pylori infection
Prebiotics	Inulin					X	X					
	FOS (includes 1-kestose)							X	X			
	GOS (includes Deshipu stachyose granules)					X	X					
	Germinated barley foodstuff								X			
	Plantago ovata seeds								X			
Probiotics	*Saccharomyces* spp.											
	S. boulardii (includes strain CNCM I-745)	X	X	X				X				X
	Lactobacillus spp.											
	L. acidophilus				X							X
	L. reuteri	X										
	L. casei (includes strain DN 114001)		X					X				
	L. rhamnosus GG	X	X		X							
	L. plantarium						X					
	Bifidobacterium spp.											
	B. lactis	X				X						
	B. infantis										X	
	Streptococcus spp.											
	S. faecium						X					
	Escherichia spp.											
	E. coli DSM 17252						X					
	Multi-strain formulations											
	VSL#3®				X				X	X		
	L. acidophilus + *B. bifidum*				X							
	L. acidophilus LAC-361 + *B. longum* BB-536				X							
	L. acidophilus CL1285 + *L. casei* LBC80R			X								
	L. plantarum LRCC5193 + *S. thermophilus* MG510					X						
	E. faecalis + *B. longum*						X					
	Lactobacillus spp + *Bifidobacterium* spp + *Saccharomyces* spp	X										
	Bacillus spp + *Enterococcus* spp + *Clostridium* spp	X										
	L. acidophilus + *B. longum* + *Enterococcus faecalis*							X				
	L. acidophilus + *L. bulgaricus* + *B. bifidum*			X								
	L. acidophilus + *B. bifidum* + *L. rhamnosus*					X						
	L. acidophilus LA-5 + *L. plantarum* + *B. lactis* BB-12 + *S. boulardii*											X
	L. acidophilus Rosell-52 + *L. acidophilus* Rosell-11 + *B. infantis* Rosell-1755 + *S. boulardii*											X
	L. acidophilus + *L. bulgaricus* + *B. bifidum* + *S. thermophilus*			X								
	L. acidophilus LA3 + *L. casei* BGP93 + *B. lactis* BLC1					X						
	L. casei, *L. plantarum*, *B. lactis*, *B. breve* Bbr8, and *B. breve* B110										X	
	L. acidophilus + *L. rhamnosus* + *B. longum* + *B. bifidum* + *B. lactis* + *S. thermophilus*						X					
	L. acidophilus + *L. rhamnosus* + *L. paracasei* + *L. casei* + *B. bifidum* + *B. longum* + *B. lactis* + *B. animalis*					X						
Synbiotics	Lactulose + *L. acidophilus*				X							
	FOS + *Bacillus coagulans*							X				
	scFOS + *B. longum W11*						X					
	Psyllium + *B. longum*								X			
	Acacia dietary fibre + *B. lactis* BB-12 + *S. thermophilus* + *L. acidophilus*						X					
	Pectin + GOS + *L. acidophilus* LA-5 + *B. lactis* BB-12 + *L. bulgaricus* + *S. thermophilus*											X
	FOS + GOS + *L. casei* + *L. rhamnosus* + *L. plantarum* + *B. lactis*					X						

*Not an exhaustive list of prebiotics or probiotics studied for GI conditions

Abbreviations: FOS, fructooligosaccharide; scFOS, short-chain fructooligosaccharide; GOS, galactooligosaccharide
