# Supplementary material for: Prebiotics and Probiotics for Gastrointestinal Disorders
Source: Nutrients. 2024 Mar 9;16(6):778. doi: 10.3390/nu16060778 (PMC10975713; doi:10.3390/nu16060778)
Supplement: Supplementary file 1 [file nutrients-16-00778-s001.zip › Supplementary Material 1.pdf]

Supplementary Material 1. Literature Search Query

((prebiotic*) OR (probiotic*) OR (synbiotic*) OR (Lactobacillus) OR (Bifidobacterium) OR (Saccharomyces) OR (ferment*) OR (kefir) OR (yogurt) OR (inulin) OR (xylooligosaccharide) OR (fructooligosaccharide) OR (galactooligosaccharide) OR (oligosaccharide) OR (resistant starch) OR (pectin) OR (beta-glucan) OR (fiber) OR (lactulose) OR (plantago ova) OR (germinated barley)) AND ((gastro*) OR (intestin*) OR (irritable bowel) OR (inflammatory bowel) OR (Crohn*) OR (ileitis) OR (enteritis) OR (colitis) OR (celiac) OR (pylori)) AND (("clinical study"[Publication Type]) OR ("observational study"[Publication Type]) OR ("clinical trial"[Publication Type]) OR ("controlled clinical trial"[Publication Type]) OR ("equivalence trial"[Publication Type]) OR ("guideline"[Publication Type]) OR ("meta-analysis"[Publication Type]) OR ("practice guideline"[Publication Type]) OR ("pragmatic clinical trial"[Publication Type]) OR ("randomized controlled trial"[Publication Type]) OR ("systematic review"[Publication Type]))
