# Supplementary figures and images for: Prebiotics and Probiotics for Gastrointestinal Disorders
Source: Nutrients. 2024 Mar 9;16(6):778. doi: 10.3390/nu16060778 (PMC10975713; doi:10.3390/nu16060778)

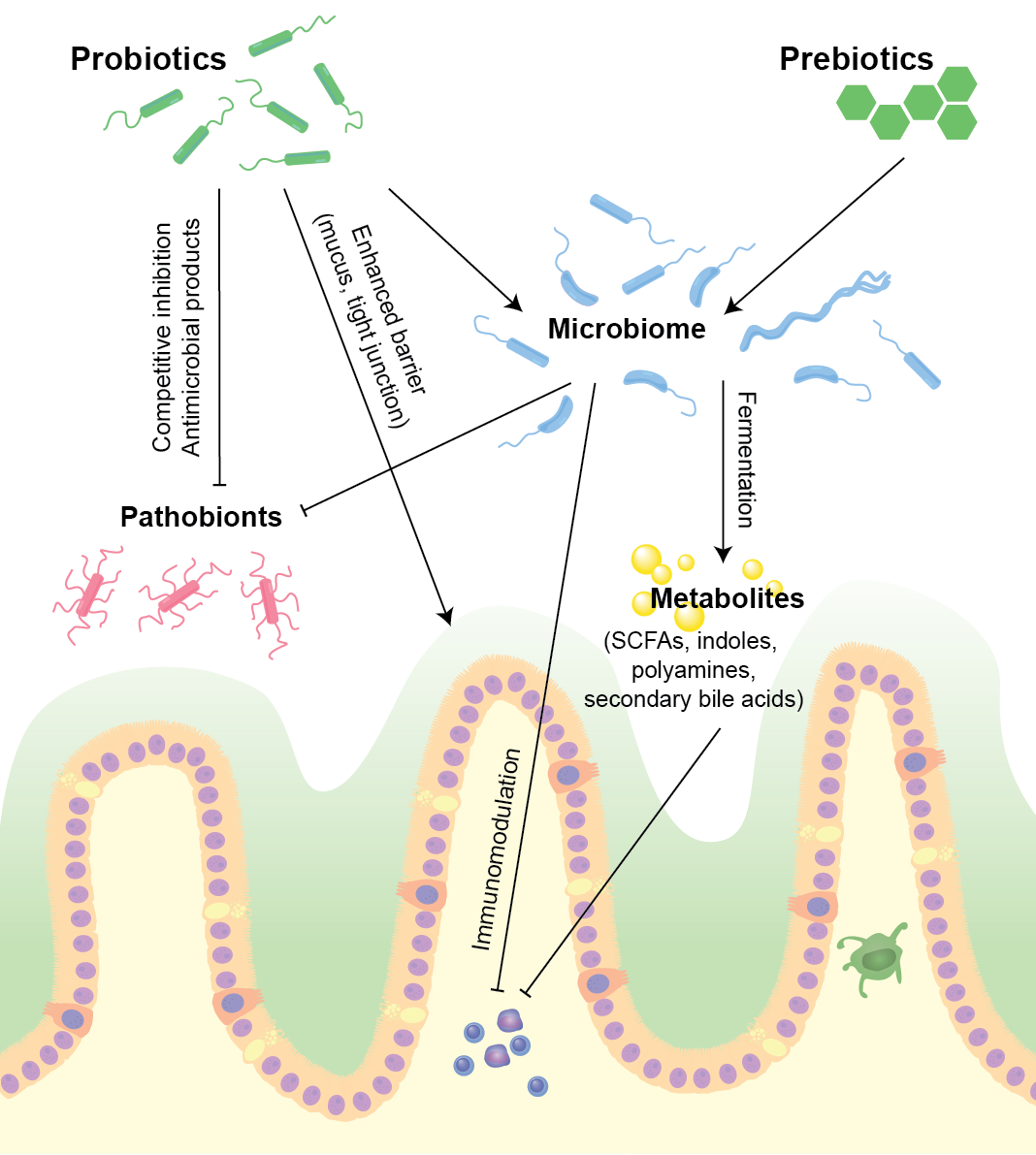

Supplement: Supplementary file 1 [file nutrients-16-00778-s001.zip › pre-probiotics microbiome v2.png]
